# Acyl Coenzyme A Synthetase Long-Chain 1 (*ACSL1*) Gene Polymorphism (rs6552828) and Elite Endurance Athletic Status: A Replication Study

**DOI:** 10.1371/journal.pone.0041268

**Published:** 2012-07-19

**Authors:** Thomas Yvert, Zi-Hong He, Catalina Santiago, Yang Hu, Yan-Chun Li, Félix Gómez-Gallego, Carmen Fiuza-Luces, Zoraida Verde, Carlos A. Muniesa, Jesús Oliván, Alfredo Santalla, Jonatan R. Ruiz, Alejandro Lucia

**Affiliations:** 1 Department of Biomedicine, European University of Madrid, Madrid, Spain; 2 Biology Center, China Institute of Sport Science, Beijing, China; 3 Science and Research Center of Beijing Sports University, Beijing, China; 4 Faculty of Sports Sciences, European University of Madrid, Madrid, Spain; 5 Faculty of Sports Sciences, Pabo de Olavide University, Seville, Spain; 6 Department of Physical Education and Sport, School of Sport Sciences, University of Granada, Granada, Spain; 7 Department of Biosciences and Nutrition at NOVUM, Unit for Preventive Nutrition, Karolinska Institutet, Stockholm, Sweden; University of Zaragoza, Spain

## Abstract

The aim of this study was to determine the association between the rs6552828 polymorphism in acyl coenzyme A synthetase (*ACSL1*) and elite endurance athletic status. We studied 82 Caucasian (Spanish) World/Olympic-class endurance male athletes, and a group of sex and ethnically matched healthy young adults (*controls*, n = 197). The analyses were replicated in a cohort of a different ethnic origin (Chinese of the Han ethnic group), composed of elite endurance athletes (runners) [*cases*, n = 241 (128 male)] and healthy sedentary adults [*controls*, n = 504 (267 male)]. In the Spanish cohort, genotype (*P* = 0.591) and minor allele (A) frequencies were similar in cases and controls (*P* = 0.978). In the Chinese cohort, genotype (*P* = 0.973) and minor allele (G) frequencies were comparable in female endurance athletes and sedentary controls (*P* = 0.881), whereas in males the frequency of the G allele was higher in endurance athletes (0.40) compared with their controls (0.32, *P* = 0.040). The odds ratio (95%CI) for an elite endurance Chinese athlete to carry the G allele compared with ethnically matched controls was 1.381 (1.015–1.880) (*P*-value = 0.04). Our findings suggest that the *ACSL1* gene polymorphism rs6552828 is not associated with elite endurance athletic status in Caucasians, yet a marginal association seems to exist for the Chinese (Han) male population.

## Introduction

Elite athletic status is a complex phenotype, with several genetic polymorphisms, many of which remain to be identified, contributing to athletic success, whether individually or in combination with other polymorphisms [1]. Genome-wide association (GWA) can help identifying novel candidate polymorphisms associated with elite endurance status. This type of studies evaluates association of genetic variation with outcomes or traits of interest by using 100,000 to 1 million or more markers across the genome without any previous hypotheses about potential mechanisms [2]. A GWA study was recently conducted by Bouchard *et al.* [3] in sedentary Caucasians to study the association of 324,611 single-nucleotide polymorphisms (SNPs) and the trainability of one of the main phenotype traits indicative of human endurance performance, i.e. maximal oxygen uptake (VO_2_max). The strongest association with the training response of VO_2_max was found to acyl coenzyme A synthetase long-chain 1 (*ACSL1*) gene polymorphism (rs6552828). The *ACSL1* gene is a candidate to explain individual variability in endurance performance, as well as in some health-related phenotypes, owing to its potential role in aerobic metabolism at the adypocite, cardiomyocite, liver and skeletal muscle fiber level [4], [5], [6], [7], [8], [9].

The findings of GWA studies should be further explored in genetic association studies focused on those SNPs showing the highest level of association [2]. Thus, the purpose of the present case:control study was to determine the association between the *ACSL1* rs6552828 polymorphism and elite endurance athletic status. To this end, we studied a cohort that comprised Caucasian (Spanish) World/Olympic-class endurance male athletes (*cases*), and sex and ethnically-matched healthy young adults (*controls*). We also studied a replication cohort of a different ethnic origin (Chinese of the Han ethnic group), composed of elite endurance athletes (*cases*), and healthy sedentary adults (*controls*). Owing to the important putative role of *ACSL1* in aerobic-related phenotypes [4], [5], [6], [7], [8], [9] we hypothesized that the *ACSL1* rs6552828 polymorphism is associated with elite endurance athletic status.

## Methods

### Participants

The research project was in accordance with the Declaration of Helsinki, it was approved by the corresponding University Review Boards [*Universidad Europea de Madrid* (UEM), Spain and China Institute of Sport Science (Beijing, China)]. Written consent was obtained from each participant. Our study adhered to most of the recent guidelines for STrengthening the REporting of Genetic Association studies (STREGA), including issues such as replication, selection of participants, rationale for choice of genes and variants, statistical methods or relatedness [10].

#### Spanish cohort

The population [n = 383, all unrelated Spanish (Caucasian for ≥3 generations) healthy males] originally comprised:

- 93 endurance elite athletes aged 19–39 years (*cases*). This sample included 22 elite endurance runners (mostly 5,000–10,000 m specialists). All had participated at least twice in previous Olympiads and 11 of them were medallists in previous World or Europe Championships. Their mean±SD VO_2_max was 78.0±1.7 ml·kg^−1^·min^−1^ (range: 69, 87). The sample also included 32 professional road cyclists who were all *Tour de France* finishers, included three top-3 finishers in this race and several stage winners in the three main tour races (*Tour*, *Giro*, *Vuelta*). Their mean±SD maximal oxygen uptake (VO_2max_) was 74.5±1.7 ml·kg^−1^·min^−1^ (62, 86). The endurance athletic cohort also comprised 39 rowers [VO_2max_: 71.7±0.8 ml·kg^−1^·min^−1^ (58, 87) or 5.2±0.1 L·min^−1^ (4.2, 6.0)] who were among *the best* in the World, i.e. each had won ≥ one bronze, silver or gold medal in the lightweight category (skip or scull, including a total of 6 gold medals) in the World Championships held during 1997–2006. The mean VO_2_max of the whole athletic cohort was 73.7±6.4 ml·kg^−1^·min^−1^.
- 197 healthy, non-athletic controls aged 19–32 years (VO_2_max: 50.2±0.3 ml·kg^−1^·min^−1^). All were undergraduate Physical Education students from the same university (UEM). Inclusion and exclusion criteria for this group were to be free of any diagnosed cardiorespiratory disease and not to be engaged in competitive sports or in formal, supervised exercise training (i.e. performing less than 3 structured weekly sessions of strenuous exercise as running, swimming, bicycling, and weight lifting) or to have a family history of competitive sports participation.

The VO_2_max values of runners, cyclists and rowers were obtained using a breath-by-breath system (Oxycon Pro System, Jaeger, Wuerzburg, Germany) in laboratory treadmill, cycle-ergometer or rower-ergometer tests performed until volitional exhaustion. The VO_2max_ of controls was estimated from the time to complete 2,000 meter tests [11]; the tests were performed inside a 400-meter outdoor track under similar environmental conditions (temperature, ∼23–24°C; relative humidity, 45–55%; barometric pressure, ∼720 mmHg).

#### Replication cohort

The population [n = 751, men and women, all unrelated of Chinese descent (Han origin) for ≥3 generations] comprised:

- 246 (131 men, 115 women) unrelated elite endurance runners aged 19 to 37 years. They were the best Chinese runners in middle-distance (1,500 m) (n = 18, 10 men, 8 women) 3,000 to 10,000 m track events (n = 164; 90 men, 74 women) and marathon in the last years (n = 64; 31 men, 33 women), including World record holders, and most of whom had participated in at least one World-championship or Olympiad.
- 505 (267 men, 238 women) healthy unrelated control participants aged 19 to 23 years [non-athletic undergraduate students from the China Agricultural University (Beijing) with no self-reported history (or family history) of competitive sports participation].

### Genotyping

Our study followed recent recommendations for replicating genotype-phenotype association studies [12]: genotyping was performed specifically for research purposes, and the researchers in charge of genotyping were totally blinded to the participants' identities [DNA samples were tracked solely with bar-coding and personal identities were only made available to the main study researcher (in Spain or China) who was not involved in actual genotyping].

#### Spanish cohort

We obtained DNA from participants' blood or saliva samples over years 2004–2008 and used the classical phenol-chloroform DNA extraction protocol with alcoholic precipitation. Genomic DNA was resuspended in 50 µl milli-Q H_2_O and stored at −20°C. Genotyping was performed during April–May 2011 in the Genetics laboratory of the UEM. Polymerase chain reaction (PCR) amplification was performed using a StepOne™ Real-Time PCR System (Applied Biosystems, Foster City, CA). Allelic discrimination analysis for the ACSL1 rs6552828 polymorphism was performed with predesigned Applied Biosystems TaqMan® SNP Genotyping Assays on demand (Assay ID: C__30469648_10).

#### Replication cohort

We obtained samples of peripheral whole blood from elite athletes (during years 2003 and 2004, and 2009–2010) and controls (2004 and 2011) and extracted genomic DNA using a Wizard Genomic DNA Purification Kit (Promega, Madison, Wisconsin, USA). Genotype analyses were performed during June–July 2011 in the Science and Research Centre of the Beijing Sports University (Beijing, China).

For high-throughput genotyping of the *ACSL1* rs6552828 polymorphism, we used a matrix-assisted laser desorption/ionization time-of-flight mass spectrometry (MALDI-TOF MS) platform (Sequenom, San Diego, CA, USA). Primers for PCR and single base extension were designed by using the Assay Designer software package (Sequenom, San Diego, CA, USA). The DNA sample was diluted to 5 ng/µl, and 1 µl of DNA was combined with 0.95 µl of water, 0.625 µl of PCR buffer containing 15 mM MgCl_2_, 1 µl of 2.5 mM dNTP, 0.325 ul of 25 mM MgCl_2_, 1 µl of PCR primers and 0.1 µl of 5units/µl HotStar Taq (Qiagen, Düsseldorf, Germany). The PCR conditions were as follows: 94°C for 15 min followed by 45 cycles at 94°C 20 s, 56°C 30 s, and 72°C 1 min, and a final incubation at 72°C 3 min. After PCR amplification, the remaining dNTPs were dephosphorylated by adding1.53 µl of water, 0.17 µl of Shrimp Alkaline Phosphatase (SAP) buffer, and 0.3 units of SAP (Sequenom, San Diego, CA, USA). The reaction was placed at 37°C for 40 min, and the enzyme was deactivated by incubation at 85°C for 5 min. Thereafter, the single base extension (SBE) over the SNP was combined with 0.755 µl of water, 0.2 µl of 10X iPLEX buffer (Sequenom, San Diego, CA, USA), 0.2 µl of termination mix, 0.041 µl of iPLEX enzyme (Sequenom, San Diego, CA, USA), and 0.804 µl of 10 µM extension primer. The SBE conditions were as follows: 94°C 30 s, 94°C 5 s, 5 cycles at 52°C for 5 s, 40 cycles at 80°C for 5 s, and final extension at 72°C for 3 min. The reaction mix was desalted by adding 6 mg of cation exchange resin (Sequenom, San Diego, CA, USA), mixed and resuspended in 25 µl of water. The completed genotyping reactions were spotted onto a 384 well Spectro Chip using a MassARRAY® Nanodispenser and determined by a matrix-assisted laser desorption ionization time-of-flight mass spectrometer (Sequenom, San Diego, CA, USA). Genotype calling was performed in real time with MassARRAY® RT software (version 3.0.0.4) and analyzed using the Massarray Typer software, version 3.4 (Sequenom, San Diego, CA, USA).

### Statistical analysis

Genotypic and allele frequencies were compared among sedentary controls and endurance athletes using the χ^2^ test. We used logistic regression analysis to analyse the association of the *ACSL1* rs6552828 polymorphism with elite endurance athletic status. The analyses were conducted in Spanish and Chinese separately, and in the case of the Chinese population, the analyses were conducted separately for men and women. All statistical analyses were performed using the PASW (v. 18.0 for WINDOWS, Chicago) and the α was set at 0.05.

## Results

### Spanish cohort

There were no failures in sample collection, DNA acquisition or genotyping procedures, except for 11 athletes, for which the amount of DNA gathered from saliva was insufficient to allow *ACSL1* rs6552828 genotype assessment. Genotype distributions met Hardy-Weinberg equilibrium (HWE) in both controls and athletes (Table 1). Genotype (*P* = 0.591) and minor allele (A) frequencies were similar in sedentary controls and athletes (*P* = 0.978). The odds ratio (OR) and 95% confidence interval (95%CI) for the association between carriage of the A allele of the *ACSL1* rs6552828 polymorphism and athletic status was 0.997 (0.819–1.214).

**Table 1:** Genotype frequencies [n(%)] of *ACSL1* rs6552828 polymorphism in Spanish controls (n = 197) and elite endurance athletes (n = 82).

	Controls	Endurance
AA	25 (12.7%)	14 (17.1%)
AG	98 (49.7%)	37 (45.1%)
GG	74 (37.6%)	31 (37.8%)
MAF	0.37	0.39
HWE	0.395	0.605

MAF, minor allele frequency (A); HWE, Hardy Weinberg Equilibrium.

Genotype: χ^2^ = 1.052, *P* = 0.591; Allele: χ^2^ = 0.001, *P* = 0.978.

### Replication cohort

There were no failures in sample collection, DNA acquisition or genotyping procedures, except for 1 participant (female) in the control group, and 5 (3 male, 2 female) in the athlete's group. Genotype distributions were in HWE except for male endurance athletes (*P* = 0.035, Table 2). In females, genotypic (P = 0.973) and allele frequencies (P = 0.881) were similar in endurance and controls, whereas in males both genotypic (P = 0.019) and allelic (P = 0.040) differed in endurance and controls. The odds ratio (95%CI) for an elite endurance Chinese athlete of having the G allele compared with ethnically-matched controls was 0.975 (0.697–1.364, P = 0.881) for females, whereas in males, the odds ratio of having the G allele was 1.381 (1.015–1.880, P = 0.04).

**Table 2:** Genotype frequencies [n(%)] of *ACSL1* rs6552828 polymorphism in Chinese controls (n = 504, 267 men, 237 women) and elite endurance athletes (n = 241, 128 men, 113 women).

	Controls	Endurance
*Males*		
AA	122 (45.7%)	40 (31.3%)
AG	115 (43.1%)	73 (57.0%)
GG	30 (11.2%)	15 (11.7%)
MAF	0.32	0.40
HWE	0.712	0.035
*Females*		
AA	102 (43.0)	49 (43.4)
AG	110 (46.4)	53 (46.9)
GG	25 (10.5)	11 (9.7)
MAF	0.33	0.33
HWE	0.560	0.539

MAF, minor allele frequency (G); HWE, Hardy Weinberg Equilibrium.

Males: Genotype: χ^2^ = 7.961, *P* = 0.019; Allele: χ^2^ = 4.226, P = 0.040.

Females: Genotype: χ2 = 0.055, *P* = 0.973; Allele: χ2 = 0.022, *P* = 0.881.

## Discussion

The main finding of our study was that the *ACSL1* rs6552828 SNP, located in the first intron of the *ACSL1* gene, 715 bp and 718 bp upstream of exon 2 and the start codon, respectively, was not associated with elite endurance athletic status in Spanish men, yet a marginal association was found in Chinese men. The *ACSL1* gene is a putative candidate to explain individual variability in endurance performance, as well as in some health related phenotypes, owing to its potential role in aerobic metabolic adaptations to regular exercise, at the adypocite, cardiomyocite, liver and skeletal muscle fiber level. Long-chain acyl coenzyme A (acyl-CoA) synthetase (ACSL) isoenzymes, of which ACSL1 is the main and most studied isoenzyme convert free fatty acids (FFA) to acyl coenzyme A (acyl-CoA) in an ATP-dependent manner, simultaneously activating and trapping FFA within cells [13]. Activation of FFA to acyl-CoA is required before FFAs can be oxidized to provide ATP. ACSL1 is highly expressed in major energy-metabolizing tissues such as fat, liver, and skeletal muscles [5], [6]. Recent research also supports evidence for an important role of ACSL1 in heart metabolism [14].

The role of ACSL1 in FFA oxidation in different tissues has been shown using transgenic mice models. Mice lacking ACSL1 specifically in adipose tissue have defects in adipose FFA oxidation [9], whereas those unable to express ACSL1 in heart ventricles show compensatory catabolism of glucose and amino acids leading to mTOR activation and cardiac hypertrophy without lipid accumulation or dysfunction [14]. In contrast, mice overexpressing ACSL1 specifically at the heart level show markedly impaired metabolic homeostasis with accumulation of triglycerides and phospholipids [15].

*ACSL1* is a candidate to explain individual differences in some several disease and endurance exercise-related phenotypes. Recent research has shown that the *ACSL1* rs9997745 polymorphism influences the risk of metabolic disease, most likely via disturbances in FFA metabolism [16]; no individual or combined association was however found for other SNPs of this gene, i.e. rs4862417, rs13120078, rs12503643 and the one we studied here, rs6552828. A GWA study recently conducted by Bouchard *et al.* [3] on 324,611 SNPs identified a set of 21 SNPs accounting for 49% of the variance in the trainability of VO_2_max [3]. The strongest association with the training response of VO_2_max was found to *ACSL1* rs6552828. In the single-SNP analyses, rs6552828 explained 6.1% of the variance in the response of VO_2_max. Homozygotes of the rs6552828 minor allele (AA) had 125 mL/min (−28%) and 63 mL/min (−17%) lower VO_2_max response than the common allele homozygotes (GG) and the heterozygotes (AG) respectively. Interestingly, in our study the A allele was less frequent in elite male endurance Chinese athletes compared with their controls. It must be also kept in mind that the A allele was the major allele in the Chinese cohort, which highlights inter-ethnic differences in genotype distributions.

To our knowledge, there is no functional data on the rs6552828 SNP; thus, we can only speculate about mechanisms underlying our findings. The intronic location of this SNP has the potential to affect mRNA stability or to modulate *ACSL1* gene transcriptional activity. Indeed, non-coding SNPs could regulate the alternative splicing of mRNA leading to changes in gene expression [17] and phenotype traits [18], [19]. Non-coding SNPs can also influence the binding of transcription factors [20]. Another possibility is that the *ACSL1* is part of the group of candidate genes, among which are calcineurin genes [21], but many of which are yet to be identified, whose cumulative effect explains, at least partly, individual variations in endurance performance in the Chinese Han population. It could also be possible that the rs6552828 SNP may be a surrogate marker for other functional *ACSL1* SNPs in the region.

We believe the results of our study are overall valid, as all the following criteria were met [2]: cases clearly presented the main study phenotype (i.e. being an elite athlete), as we studied some of the best elite endurance athletes world-wide, participants within both cohorts were ethnically-matched, genetic assessment was accurate and unbiased, genotype distributions were in HWE in the control group of the two cohorts, and we used a replication cohort of a different ethnic origin. Current body of knowledge on genetic factors associated with exercise phenotypes and athletic status comes mainly from research performed on Caucasian populations. Further investigations are thus needed with other ethnic groups and populations as the one studied here, i.e. representing an important fraction of the total planet population.

In summary, our findings suggest that the *ACSL1* gene polymorphism rs6552828 is marginally associated with male elite endurance status in Chinese (Han) population yet such association was not found in Chinese females or in a different (Caucasian) cohort. Our findings exemplify the need for further genetic association studies in the field of sport sciences to use at least two cohorts of a different ethnic background in order to increase the generalisability of their results.
