# Correction: Acyl Coenzyme A Synthetase Long-Chain 1 (*ACSL1*) Gene Polymorphism (rs6552828) and Elite Endurance Athletic Status: A Replication Study

**DOI:** 10.1371/journal.pone.0093166

**Published:** 2014-03-17

**Authors:** The PLOS ONE Staff

It has come to our attention that the Academic Editor who handled this manuscript, Dr Nuria Garatachea, has collaborated in recent publications with the authors of the article.

In line with the *PLOS ONE* competing interests policy (http://www.plosone.org/static/competing.action), we consider this as a potential conflict of interest.

In the light of this potential competing interest, the *PLOS ONE* editors have asked an independent member of the editorial board to carefully evaluate the peer review process of this article. This adviser considered that additional clarification should be supplied in relation to some of the methodological aspects of the study. The authors have provided the requested clarifications in relation to the Methods and Discussion sections, which they report as part of this Correction:

The study made use of a convenience Spanish cohort sample and no power calculation was done at the time of recruitment, the post-hoc power calculation for this study is 0.394. As stated in the paper all athletes and controls were either Caucasian (Spanish) or Chinese (of Han origin) for ≥3 generations.

The authors acknowledge that a limitation of the present study (and in fact of most sports genetics studies) is the lack of ancestry informative markers to validate the matching on ethnicity and thus to rule out the possibility of population substructure potentially confounding the results. Thus, reporting ancestry informative markers would be suitable in future publications in the field of sports genetics.
